# Detection and Characterization of New Coronavirus in Bottlenose Dolphin, United States, 2019

**DOI:** 10.3201/eid2607.200093

**Published:** 2020-07

**Authors:** Leyi Wang, Carol Maddox, Karen Terio, Saraswathi Lanka, Richard Fredrickson, Brittany Novick, Celeste Parry, Abby McClain, Kyle Ross

**Affiliations:** University of Illinois, Urbana, Illinois, USA (L. Wang, S. Lanka, R. Fredrickson);; University of Illinois College of Veterinary Medicine, Urbana (C. Maddox);; University of Illinois College of Veterinary Medicine, Brookfield, Illinois, USA (K. Terio);; National Marine Mammal Foundation, San Diego, California, USA (B. Novick, C. Parry, A. McClain);; Naval Information Warfare Center Pacific, San Diego (K. Ross)

**Keywords:** coronavirus, viruses, detection, genetic characterization, bottlenose dolphin, zoonoses, United States

## Abstract

We characterized novel coronaviruses detected in US bottlenose dolphins (BdCoVs) with diarrhea. These viruses are closely related to the other 2 known cetacean coronaviruses, Hong Kong BdCoV and beluga whale CoV. A deletion in the spike gene and insertions in the membrane gene and untranslated regions were found in US BdCoVs (unrelated to severe acute respiratory syndrome coronavirus 2).

The coronavirus family consists of single-stranded, positive-sense RNA viruses that cause respiratory, gastrointestinal, hepatic, and neurologic diseases of different host species. On the basis of genetic characterization, coronaviruses have been classified into 4 genera: *Alphacoronavirus*, *Betacoronavirus*, *Gammacoronavirus*, and *Deltacoronavirus*. *Cetacean coronavirus* is a recently proposed new species in the genus *Gammacoronavirus*, in addition to a common species (*Avian coronavirus*) (*1*). *Cetacean coronavirus* species contains bottlenose dolphin coronavirus (BdCoV) HKU22, identified in 2014, and beluga whale coronavirus (BWCoV) SW1, identified in 2008 (*1*,*2*). We report detection and genetic characterization of BdCoV in bottlenose dolphins in the United States; all dolphins had clinical signs consistent with gastrointestinal discomfort.

Four Atlantic bottlenose dolphins cared for by the US Navy Marine Mammal Program (San Diego, CA) showed development of an acute onset of clinical illness with clinical signs consisting of inappetence (n = 4), diarrhea (n = 3), and lethargy (n = 2) during April and May 2019. We collected fecal samples as part of the minimum workup for acute illness. Among all viruses we tested by using conventional PCRs, only coronavirus showed a positive result for all 4 dolphins.

We further evaluated samples by using next-generation sequencing as described (*3*). De novo assembly analysis of raw FASTQ data showed that 4 near complete genomes of BdCoV were assembled. Gaps were closed by Sanger sequencing at ACGT, Inc. (https://www.acgtinc.com). The genomes of all 4 US BdCoVs (37112–1, −2, −3, and −4) comprised 31,728 nt (GenBank accession nos. MN690608–11), which were shorter than those of 3 Hong Kong BdCoVs (HK-BdCoVs) (31,750–31,758 nt).

Further analysis of all individual genes showed that the 4 US BdCoV strains showed similar identities to both HK-BdCoVs and BWCoV in open reading frame (ORF) 1a, ORF1b, nonstructural (NS) 7, NS8, NS9, and NS10. However, US BdCoV strains showed higher identities to HK-BdCoVs than to BWCoV only in spike (S), envelope (E), membrane (M), and NS5a instead of all remaining genes (Appendix Table, Figure 1).

The 4 US BdCoV strains showed relatively higher identities to BWCoV than to HK-BdCoVs in NS5b (95.9% vs. 93.8%–94.0%), NS5c (98.4% vs. 97.7%–97.9%), NS6 (94.9% vs. 88.6%–88.9%), and nucleocapsid protein (97.9% vs. 96.1%–96.5%) genes. Analysis of amino acid identities of different ORFs also showed similar patterns (Appendix Table).

Phylogenetic analysis of complete genomes showed that the 4 US BdCoVs were clustered with 3 HK-BdCoVs strains and distantly related to BdCoV SW1 but were distinct from avian coronaviruses (Figure). Phylogenetic trees for individual genes showed that US BdCoV strains have a greater correlation with HK-BdCoVs than BWCoV in the S, E, M, NS5a, and NS7 genes and to BWCoV than HK-BdCoVs in remaining genes (Figure; Appendix Figures 2, 3).

**Figure:**
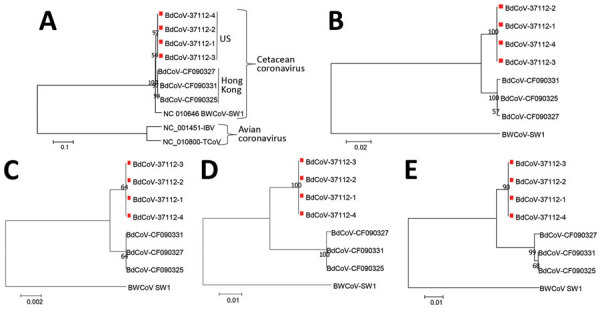
Phylogenetic analysis of A) complete genome, B) spike C) envelope, D) matrix, and E) nonstructural protein 5a genes of gammacoronaviruses, including 4 US BdCoVs, 37112–1 to −4 (GenBank accession nos. MN690608–MN690611, indicated with red squares); 3 Hong Kong BdCoVs (accession nos.: CF090327, KF793825; CF090331, KF793826; CF090325, KF793824); and 1 BWCoV (SW1, accession no. NC_010646). Numbers along branches are bootstrap values. Scale bars indicate nucleotide substitutions per site. BdCoV, bottlenose dolphin coronavirus; BWCoV, beluga whale coronavirus.

Compared with the Hong Kong CF090331 strain, all US BdCoV strains have a 42-nt deletion in the S1 region at positions 21366–21407 encoding an S protein that is 14-aa shorter (Appendix Figure 4). Compared with 3 HK-BdCoVs strains, a 3-nt insertion (ACA) at positions 25417–25419 was found in the M gene of the 4 US BdCoVs, leading to a frameshift mutation in the M protein that was 1 amino acid longer (Appendix Figure 5).

In addition, a 4-nt insertion (TATA) was found in the 5′ untranslated region (UTR) of US BdCoV strains, and a 1-nt insertion (T) was found in the 3′ UTR of US BdCoV strains (Appendix Figure 6). Similar to findings of a previous report (*1*), because of 1 nt mutation (G→T at position 28268) of the US BdCoV strains, a premature stop codon in the NS7 gene resulted in an NS7a (42 aa) and an NS7b (117 aa). The position of the premature stop codon in the US BdCoVs was different from that for 2 HK-BdCoVs (CF090325 and CF090331), which encode different sizes of NS7a (63 aa) and NS7b (34 aa) proteins.

S deletion variants are commonly observed in coronaviruses. Porcine respiratory coronavirus is an S gene deletion mutant of transmissible gastroenteritis virus and causes a respiratory disease instead of gastroenteritis in pigs because of a large deletion (>600 nt) in the N terminal of the S gene (*4*). In addition, a large deletion (591 nt) in S1 resulted in a change in virulence of porcine epidemic diarrhea virus (*5*). In our study, we observed that 4 US BdCoVs had a 42-nt deletion in the S1 gene. It is unclear whether this deletion region is related to the viral tropism and virulence, and warrants further studies.

During a surveillance study in Hong Kong, China, BdCoV was identified only in fecal samples from dolphins that had no notable clinical signs (*1*). In our study, genetically related BdCoVs were detected in dolphins that had diarrhea, lethargy, and inappetence in the United States. It is possible that unique genetic features of US BdCoVs, including a sequence deletion in the S gene and an insertion in the M gene and 5′ and 3′ UTRs, and mutations in different genes might have contributed to the observed clinical diarrhea signs in US dolphins. Additional surveillance is needed to monitor the evolution of this virus worldwide.

Appendix Additional information on detection and characterization of new coronavirus in bottlenose dolphin, United States, 2019.
