# Supplementary material for: Detection and Characterization of New Coronavirus in Bottlenose Dolphin, United States, 2019
Source: Emerg Infect Dis. 2020 Jul;26(7):1610–2. doi: 10.3201/eid2607.200093 (PMC7323548; doi:10.3201/eid2607.200093)
Supplement: Appendix — Additional information on detection and characterization of new coronavirus in bottlenose dolphin, United States, 2019. [file 20-0093-Techapp-s1.pdf]

Article DOI: https://doi.org/10.3201.eid2607.200093

Detection and Characterization of New Coronavirus in Bottlenose Dolphin, United States, 2019

Appendix

Cetacean Coronavirus

The major differences between HKU22 and SW1 were found in their spike (S) and membrane genes; only 74.3%–74.7% and 85.5% amino acid identities, respectively (*1*). Beluga whale coronavirus (BwCoV) SW1 was detected in liver tissue from a deceased whale, and bottlenose dolphin CoV (BdCoV) HKU22 was detected during routine surveillance study in which no dolphins showed any notable signs. Because of having unique open reading frames (ORFs) (nonstructural [NS]5a, NS5b, NS5c, NS6, NS7, NS8, NS9, and NS10) between membrane and nucleocapsid genes, both bottlenose dolphin CoV HKU22 and beluga whale CoV SW1 have the largest genome sizes among all CoVs.

Animal Care

Fecal samples were collected at the US Navy Marine Mammal Program (MMP) during routine care of dolphins and under US Code, Title 10, USC 7524. The MMP houses and cares for a population of bottlenose dolphins in San Diego Bay (CA, USA). MMP is accredited by the Association for Assessment and Accreditation of Laboratory Animal Care International and adheres to the national standards of the US Public Health Service Policy on the Humane Care and Use of Laboratory Animals and the Animal Welfare Act. The Navy Marine Mammal Program is accredited by the Association for Assessment and Accreditation of Laboratory Animal Care and adheres to the national Humane Care and Use of Laboratory Animals and the Animal Welfare Act. As required by the Department of Defense, the Program’s animal care and use program is routinely reviewed by an Institutional Animal Care and Use Committee and the Department of Defense Bureau of Medicine.

Viral Testing

Fecal samples of all dolphins were tested by using conventional PCR methods. Samples showed negative results for paramyxovirus, rotavirus, canine distemper virus, canine parvovirus, and adenovirus (Athens Veterinary Diagnostic Laboratory, University of Georgia, Athens, GA, USA).

Bacterial Testing

In addition to those viral testing listed in the main text, 4 dolphins were tested for different bacteria (*Campylobacter*, *Salmonella*, and *Lawsonia* species) and toxins of *Escherichia coli* (attaching and effacing toxin, cytolethal distending toxin, and cytotoxic necrotizing factor 1) and *Clostridium perfringens* (alpha toxin, enterotoxin, and beta 2 toxin). Results showed that 2 dolphins were positive for *E. coli* cytotoxic necrotizing factor 1, and 1 dolphin was positive for alpha toxin and entero toxin of *C. perfringens*.

Next-Generation Sequencing

Nucleic acids extracted from the fecal samples were subjected to a sequence-independent, single-primer amplification and library preparation by using a Nextera XT Kit (https://www.illumina.com) and sequencing by using MiSeq Reagent Kit version 2 (Illumina) (500 cycles) as described (*2*). Raw sequence data were submitted to the Sequence Read Archive under BioProject no. PRJNA612626.

Results

Raw FASTQ (Illumina) data for 4 dolphin samples were analyzed by using Kraken 2 and a standard Kraken database (*3*). Analyses showed that 99%–100% of viral reads of all 4 samples (37112–1, –2, –3, and –4) are CoV reads, and no other viruses previously tested by conventional PCR were observed. The genome organization and the transcription regulatory sequence motif 5′-AAACA of US BdCoVs are the same as those for Hong Kong BdCoVs and BdCoV SW1 (Appendix Figure 1). Alignment analysis of the complete nucleotide genome showed that 4 BdCoV strains (37112–1, –2, –3, and –4) were closely related to each other, had high identities (99.9%), and showed 97% and 94% identities with 3 Hong Kong BdCoVs and BdCoV SW1 strain, respectively, but showed low identities (46%) with avian CoVs. On the basis of the 90%

amino acid identity threshold for a new species, 4 US BdCoVs belong to the species *Cetacean CoV*.

Compared with the Hong Kong CF090331 strain, the area of the S deletion in US BdCoV seems to be a variable region. However, the Hong Kong strain CF090325 has a 9-nt deletion, and Hong Kong strain CF090325 has a 12-nt deletion.

References

1. Woo PC, Lau SK, Lam CS, Tsang AK, Hui SW, Fan RY, et al. Discovery of a novel bottlenose dolphin coronavirus reveals a distinct species of marine mammal coronavirus in Gammacoronavirus. J Virol. 2014;88:1318–31. https://doi.org/10.1128/JVI.02351-13 PubMed

2. Wang L, Stuber T, Camp P, Robbe-Austerman S, Zhang Y. Whole-genome sequencing of porcine epidemic diarrhea virus by Illumina MiSeq platform. In: Wang L, editor. Animal coronaviruses. Springer protocols handbooks. Totowa (NJ): Humana Press; 2016. p. 201–8.

3. Wood DE, Lu J, Langmead B. Improved metagenomic analysis with Kraken 2. Genome Biol. 2019;20:257. PubMed https://doi.org/10.1186/s13059-019-1891-0

Appendix Table. Nucleotide and amino acid identities of US BdCoV strains (37112–1 to –4) with other marine mammal CoVs, including 3 Hong Kong BdCoVs and 1 Bw CoV SW1*

	Nucleotide identity, %				Amino acid identity, %			
	Hong Kong BdCoVs			Bw CoV	Hong Kong BdCoVs			Bw CoV
Gene	CF090325	CF090327	CF090331	SW1	CF090325	CF090327	CF090331	SW1
ORF								
1a	97.8	97.9	97.9	97.8	97.9–98.0	98.2	98.1	98.2–98.3
1b	98.2	98.4	98.2–98.3	98.3–98.4	99.2–99.3	99.4–99.5	99.3–99.4	99.1–99.2
S	96.4	96.4	96.1	75.9	96.7	96.4	96.1	74.8
E	99.3	99.3	99.3	95.1	98.9	98.9	98.9	96.8
M	93.6	93.3	93.6	83.5	96.0	95.2	96.0	85.1
NS5a	97.1	97.1	97.1	88.7	98.5	98.5	98.5	92.0
NS5b	93.8	94.0	93.8	95.9	93.0	92.4	93.0	95.3
NS5c	97.7	97.9	97.7	98.4	97.7	98.2	97.7	97.7
NS6	88.9	88.6	88.9	94.9	91.6	91.6	91.6	95.1
NS7	94.6	94.8	94.6	94.2	–	–	–	–
NS8	97.7	97.2	97.7	97.7	96.6	94.9	96.6	96.6
NS9	98.0–98.2	97.8–98.0	98.0–98.2	98.0–98.2	96.7–97.3	96.7–97.3	96.7–97.3	96.7–97.3
NS10	99.0	98.2	99.0	98.4	99.0	97.1	99.0	99.0
N	96.1	96.5	96.4	97.9	96.0	97.0	97.0	99.7

*BdCoV, bottlenose dolphin coronavirus; BwCoV, Beluga whale coronavirus, E, envelope; M, membrane; N, nucleocapsid; NS, nonstructural; ORF, open reading frame; –, amino acid identity of NS7 gene was not determined because of a premature stop codon at different locations between US BdCoVs and Hong Kong BdCoVs.

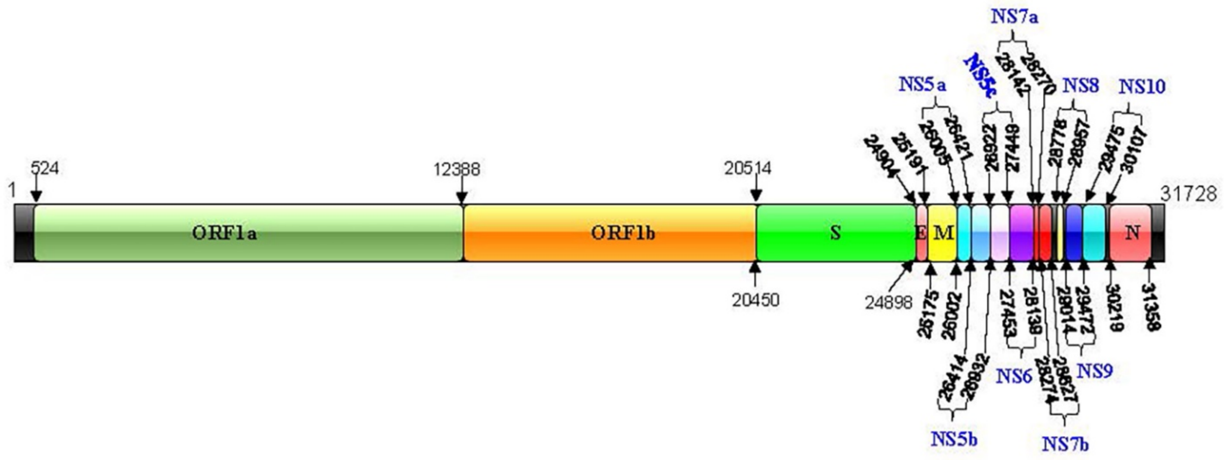

Appendix Figure 1. Schematic diagram of bottlenose dolphin coronavirus genome of US strains (37112–1 to –4). E, envelope; M, membrane, N, nucleocapsid; NS, nonstructural; ORF, open reading frame; S, spike.

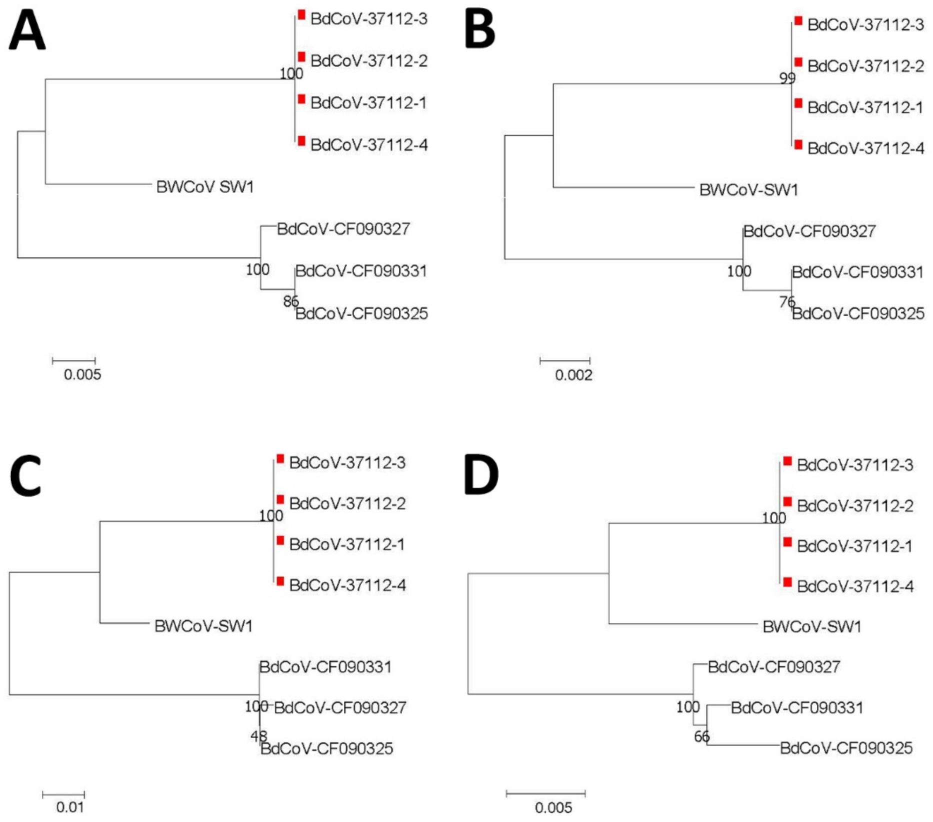

Appendix Figure 2. Phylogenetic tree analysis of NS5b, NS5c, NS6, and N genes of gammacoronaviruses, including 4 US BdCoVs (GenBank accession nos. MN690608–11, indicated by a red square), 3 Hong Kong BdCoVs (CF090327: KF793825; CF090331: KF793826; CF090325:

KF793824), and 1 BwCoV (SW1: NC_010646). A) NS5b, B) NS5c, C)NS6, D) N. Scale bars indicate nucleotide substitutions per site. BdCoV, bottlenose dolphin coronavirus; BwCoV, Beluga whale coronavirus, N, nucleocapsid; NS, nonstructural.

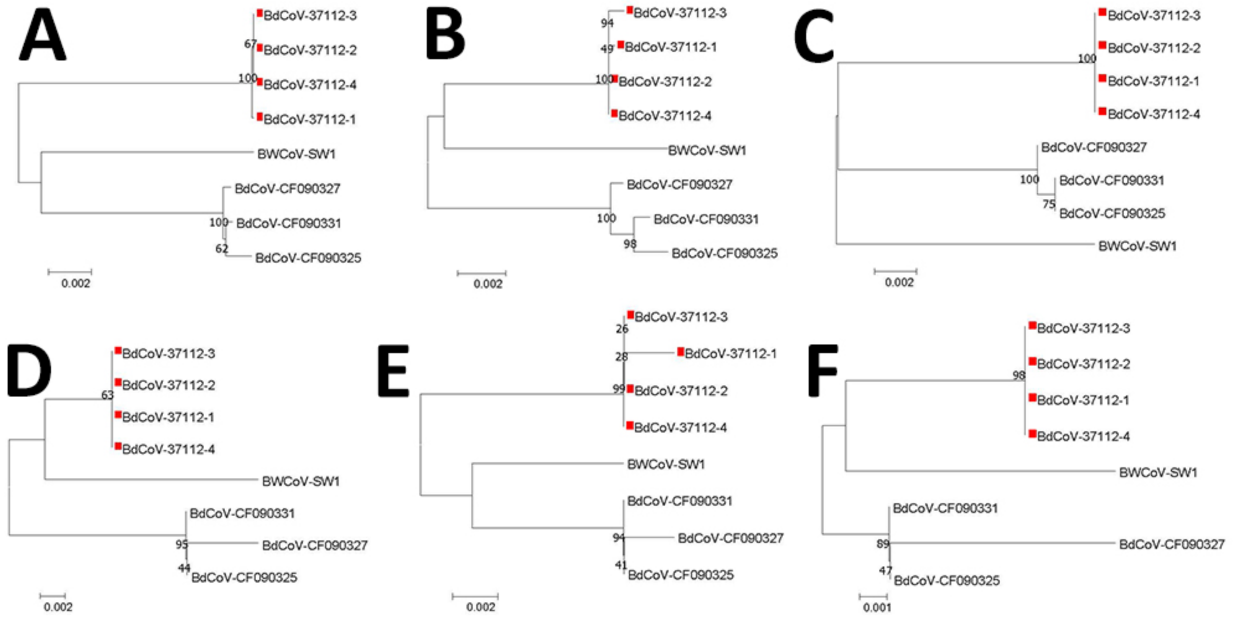

Appendix Figure 3. Phylogenetic tree analysis of A)ORF1a, B) ORF1b, C) NS7, D) NS8, E)NS9, and F) NS10 genes of gammacoronaviruses, including 4 US. BdCoVs) (GenBank accession nos. MN690608–11, indicated with a red square), 3 Hong Kong BdCoVs (CF090327: KF793825; CF090331: KF793826; CF090325: KF793824), and 1 BwCoV (SW1: NC_010646). Scale bars indicate nucleotide substitutions per site. BdCoV, bottlenose dolphin coronavirus; BwCoV, Beluga whale coronavirus, ORF, open reading frame; NS, nonstructural.

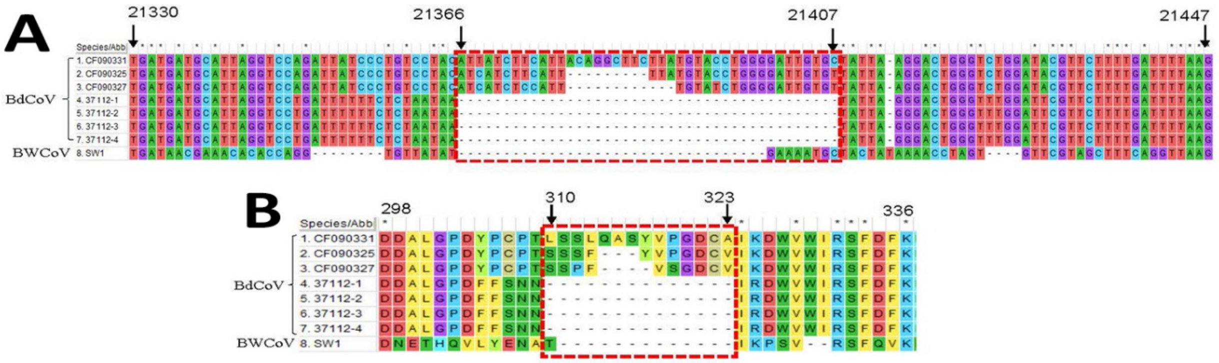

Appendix Figure 4. Nucleotide and amino acid sequence alignment of partial S gene of coronaviruses at positions 21330–21447 by using MUSCLE in MEGA 7.0.26 (https://www.megasoftware.net). Deletion region is indicated by a red color frame between positions 21366 and 21407 of the nucleotide sequence and between positions 310–323 of amino acid sequence. Labeled positions were based on CF090331 (GenBank accession no. KF793826). *, same nucleotide or amino acid; –, deletion. BdCoV, bottlenose dolphin coronavirus; BwCoV, Beluga whale coronavirus; S, spike.

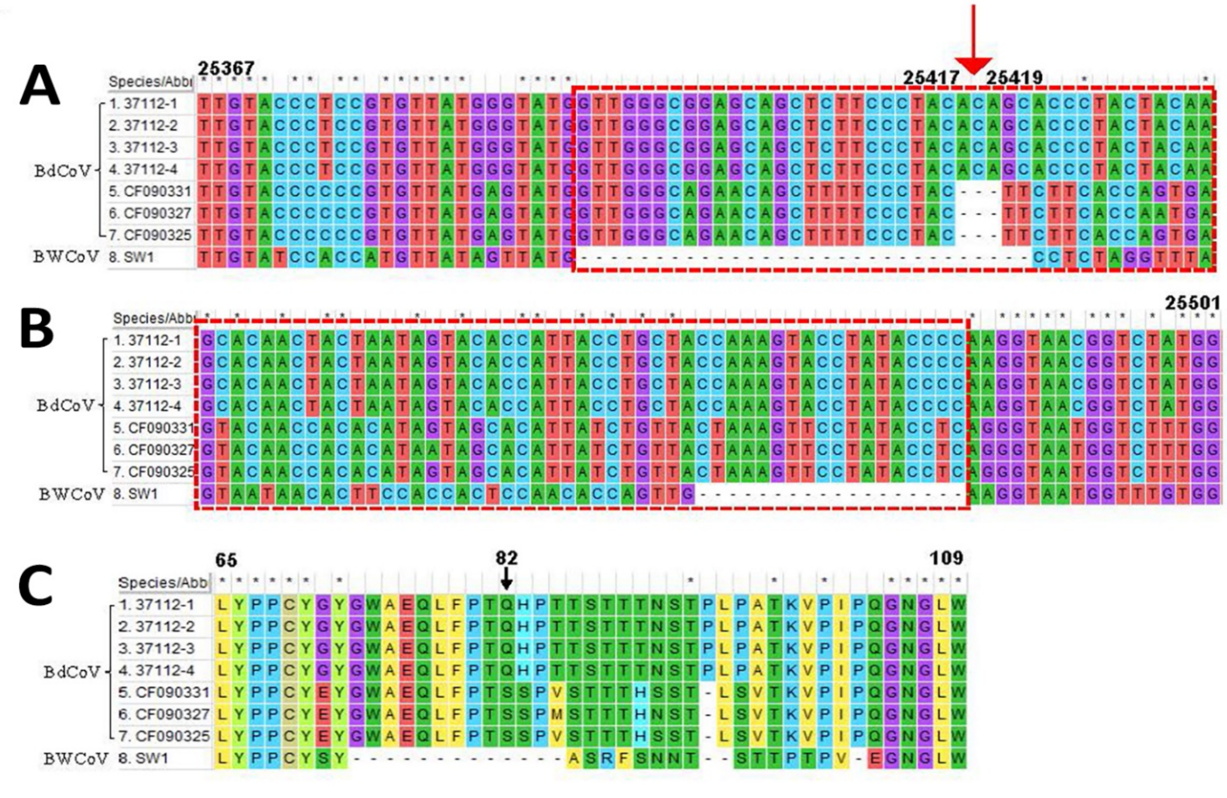

Appendix Figure 5. Nucleotide and amino acid sequence alignment of partial M gene of coronaviruses at positions 25367–25501 by using MUSCLE in MEGA 7.0.26 (https://www.megasoftware.net). Insertion region was between positions 25417 and 25419 of the nucleotide sequence, which caused frameshift

mutations starting at amino acid sequence position 82. Labeled positions were based on 37112–1 (GenBank accession no. MN690608). Red arrow indicates area/region that has a 3-nt insertion in US BdCoVs, and red boxes indicate variable area/region in the M gene in Cetacean CoV. *, same nucleotide or amino acid; –, deletion. BdCoV, bottlenose dolphin coronavirus; BwCoV, Beluga whale coronavirus; M, membrane.

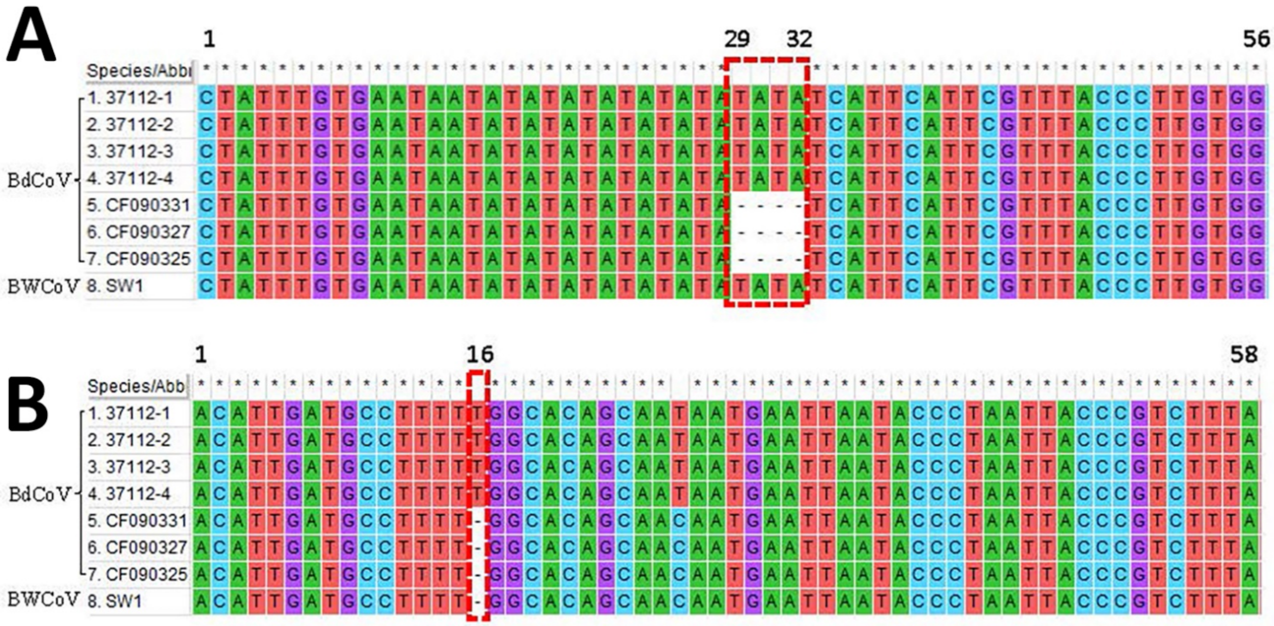

Appendix Figure 6. Nucleotide sequence alignment of partial 5′ and 3′ regions of the UTR gene of coronaviruses at positions 1–56 and 1–58, respectively, by using ClustaW in MEGA 7.0.26 (https://www.megasoftware.net). Insertion region is indicated by a red color frame at positions 29–32 and 16 of the 5′ UTR and 3′ UTR, respectively. Labeled positions were based on 37112–1 (GenBank accession no. MN690608) at different locations between US BdCoVs and Hong Kong BdCoVs. *, same nucleotide or amino acid; –, deletion. BdCoV, bottlenose dolphin coronavirus; BwCoV, Beluga whale coronavirus; UTR, untranslated region.
